# Donor Kidneys with an Anatomic Anomaly Used for Kidney Transplantation

**DOI:** 10.34067/KID.0000000000000270

**Published:** 2024-01-25

**Authors:** Nikhil A. Reddy, Sridhar R. Allam, Ashraf I. Reyad

**Affiliations:** 1 North Texas Division, HCA Healthcare Research Institute, Fort Worth, Texas; 2 Transplant Nephrology, PPG Health, Fort Worth, Texas; 3 Transplant Surgery, Medical City Fort Worth Transplant Institute, Fort Worth, Texas

**Keywords:** horseshoe kidney, renal transplantation, organ utilization

## Abstract

**Figure:**
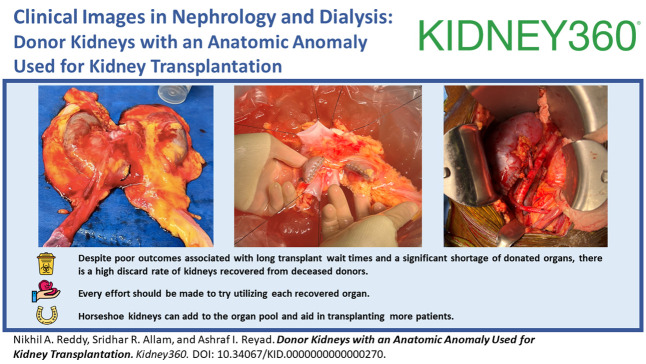

## Case Description

**Figure 1:**
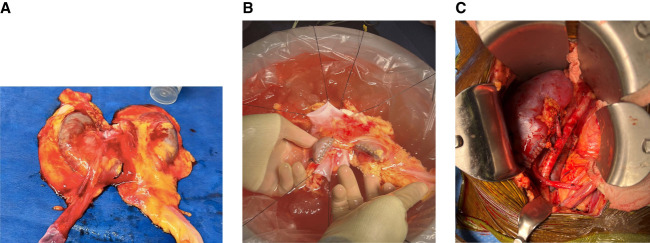
**Horseshoe kidney transplantation.** (A) Horseshoe kidney with each moiety about 9 cm in size and fused at inferior poles by a 3-cm wide isthmus. (B) Horseshoe kidney moieties were separated by dividing the isthmus using a 60-mm blue gastrointestinal anastomosis stapler and oversewn using a 5–0 chromic gut suture. (C) Immediately post-transplant, each horseshoe kidney moiety reperfused well with no bleeding at the divided site.

A 23-year-old woman with no known medical history died of a motorcycle accident. The donor's family consented for organ donation after the circulatory death. The warm ischemia time was 45 minutes. The kidney donor profile index was 19% with terminal serum creatinine of 0.8 mg/dl. Abdominal imaging revealed an incidental finding of a horseshoe kidney. This was offered as *en bloc* to one of the candidates at our center. We noted that each moiety was 9 cm in size and fused at the lower poles by a 3-cm wide isthmus (Figure 1A). A retrograde ureterogram confirmed the visual separation of renal collecting systems with no evidence of obstructive uropathy. The kidney was split using a 60-mm blue gastrointestinal anastomosis stapler and oversewn with a 5–0 chromic gut suture (Figure 1B). Meticulous back table preparation was done to transplant two different patients (Figure 1C). The first recipient is a 61-year-old man with ESKD due to diabetes mellitus who had been on hemodialysis for about 3 years. The second recipient is a 73-year-old woman with ESKD due to hypertension who had been on hemodialysis for about 4 years. Both recipients did well post-transplant with immediate kidney function and no complications, such as bleeding, urine leak, or transplant hydronephrosis, noted.

## Discussion

Horseshoe kidneys are the most common renal fusion anomaly with an estimated incidence of approximately in one in 2000 per autopsy data.^1^ They are often asymptomatic and are incidentally diagnosed on abdominal imaging. Vascular and urological abnormalities, such as ureteropelvic junction obstruction, are frequently observed in individuals with horseshoe kidneys.^2^ The utilization of horseshoe kidneys for transplantation has been reported since the 1970s.^3^ Although there are no published data on transplant center practices regarding the utilization of horseshoe kidneys, it seems that this organ pool is not widely used. In our case, this kidney offer was denied for 81 candidates on the wait list at other transplant centers before being accepted by our center at sequence number 82 on the organ offer match run. The most common denial reason given by transplant centers was organ anatomical damage or defect. Currently, more than 100,000 patients are on the organ transplant wait list in the United States. Transplant wait list times remain long and are associated with poor outcomes: 34.6% of patients remain waiting for a transplant 3 years later, and 26.4% die or are removed from the wait list.^4^ Despite these data, 25% of kidneys recovered from deceased donors are discarded.^5^ Reducing discard rates of recovered organs is critical in transplanting more patients on the wait list. Horseshoe kidneys are often avoided because of the associated vascular and urological abnormalities. With detailed examination and diligent back table preparation, a horseshoe kidney can be transplanted as *en bloc* in one recipient or, if anatomically feasible, split to transplant two recipients.

## Teaching Points

- Despite poor outcomes associated with long transplant wait times and a significant shortage of donated organs, there is a high discard rate of kidneys recovered from deceased donors.
- Every effort should be made to try utilizing each recovered organ.
- Horseshoe kidneys can add to the organ pool and aid in transplanting more patients.

## References

[1] Mcdonald JH Mcclellan DS. Crossed renal ectopia. Am J Surg. 1957;93(6):995–1002. doi:10.1016/0002-9610(57)90680-3 13424850

[2] Cascio S Sweeney B Granata C Piaggio G Jasonni V Puri P. Vesicoureteral reflux and ureteropelvic junction obstruction in children with horseshoe kidney: treatment and outcome. J Urol. 2002;167(6):2566–2568. doi:10.1016/s0022-5347(05)65038-0 11992090

[3] Majeski JA Alexander JW First R Munda R Fidler JP. Transplantation of a horseshoe kidney. JAMA. 1979;242(10):1066. doi:10.1001/jama.1979.03300100044023 381694

[4] Lentine KL Smith JM Hart A, . OPTN/SRTR 2020 annual data report: kidney. Am J Transplant. 2022;22(suppl 2):21–136. doi:10.1111/ajt.16982 35266618

[5] Mohan S Yu M King KL Husain SA. Increasing discards as an unintended consequence of recent changes in United States kidney allocation policy. Kidney Int Rep. 2023;8(5):1109–1111. doi:10.1016/j.ekir.2023.02.1081 37180509 PMC10166727

